# Targeted therapies for cancer

**DOI:** 10.1186/s12916-022-02287-3

**Published:** 2022-03-11

**Authors:** Zhijun Zhou, Min Li

**Affiliations:** Department of Medicine, Department of Surgery, the University of Oklahoma Health Sciences Center, 975 NE 10th Street, BRC 1262A, Oklahoma City, OK 73104 USA

## Abstract

Targeted therapy is the key for improving overall survival while decreasing the undesirable adverse effects of cancer treatment. Patients who received matched targeted therapies showed dramatically improved overall survival (OS) and progression-free survival (PFS) compared to those without matched therapies. However, each patient responds to targeted therapy differently due to their unique genomic profile. The discrepancy of treatment response between clinical trials and real-world clinical practice highlights an unmet need to develop tailored therapies for individual patients. The development of cutting-edge technologies, such as next-generation sequencing, has enabled us to identify more actionable targets. In this special issue of *BMC Medicine*, a collection of highly translational and clinical oncology papers presented a series of studies on targeted therapies for a variety of cancer types, aiming to bridge the gap between genomic testing and precision medicine and spark innovations on improving the efficacy of targeted therapies.

## Background

The past few decades have seen extraordinary progress in developing novel treatment options that target tumors with specific molecular perturbations. These novel treatment options, known as targeted therapies, have shifted the treatment paradigm for a subset of cancer types, such as lung cancer. Emerging evidence has supported the rationale of the combination of targeted therapies with traditional therapies or immunotherapy to achieve optimal benefit while limiting the undesirable side effects. It takes a village to develop an approved targeted therapy from scratch. However, most cancer patients were unaware of the eligibility for targeted therapies upon diagnosis, which might have otherwise improved the overall prognoses. The development of next-generation sequencing has demonstrated a valuable asset for tailoring individualized treatment strategy. Nowadays, more and more patients could benefit from these targeted therapies. Even though, treatment resistance will inevitably develop, highlighting an urgent need to develop more innovative therapies that could target the evolving vulnerabilities.

## Targeted therapy: an evolving paradigm

The evolving paradigm for the treatment of non-small-cell lung cancer (NSCLC) has provided a promising blueprint for developing novel therapeutic strategies for cancer. This collection starts with 3 clinical studies focusing on targeted therapies in NSCLC. Zhao et al. evaluated the role of genomic profiling in screening potential candidates for targeted therapy in NSCLC. They found that patients receiving matched targeted therapies showed much better outcomes than those receiving non-matched therapies [1]. Tyrosine kinase inhibitors (TKIs) have revolutionized the treatment for NSCLC, the mortality of which has been decreasing steadily, partially due to the development of these targeted therapies. Crizotinib, an inhibitor that targets ALK and ROS1, has been approved by the US Food and Drug Administration (FDA) for the treatment of NSCLC. Zhang et al. found that crizotinib presented a more dramatic anti-tumor effect in ROS1-rearranged NSCLC compared to chemotherapy. Patients with only ROS1 fusion showed better outcomes compared to those with concurrent driver mutations or concomitant mutations. Those with intracranial-only progression had more favorable prognoses than those with extracranial-only progression [2]. Treatment for patients with intracranial metastases remains an urgently unmet need, as the blood-brain barrier prevents the accessibility of most drugs. Zou et al. evaluated the efficacy of alectinib, an ALK inhibitor that can penetrate the blood-brain barrier. Alectinib showed promising efficacy in ALK-positive NSCLC patients with intracranial metastases [3]. This multicenter study demonstrated that ALK-positive NSCLC patients with brain metastases could benefit from alectinib treatment. These results provide a strong rationale for genomic testing to identify targetable genomic alterations or rearrangements.

The next study is from Dr. Ramagopalan’s group in Switzerland, which reinforced that the implementation of targeted therapy and immunotherapy have contributed to improved prognoses in patients with NSCLC [4]. The improved survival in ALK-positive tumors is associated with the development of ALK inhibitors, while the improved survival in non-oncogene-positive tumors is likely attributed to the incorporation of immunotherapy, an emerging treatment strategy that has drawn substantial attention in recent years. Notably, little if any improvement has been achieved in terms of survival in the past decade among NSCLC patients with EGFR-positive tumors. To further boost the efficacy of EGFR-TKI, Zhang and colleagues developed a novel treatment strategy which incorporated bevacizumab, an anti-angiogenesis reagent, and the first generation of EGFR-TKI (A+T) in NSCLC. They found that the A+T group had better outcomes than the T group. They explored the potential biomarkers that were correlated to treatment response and established a nomogram computational algorithm to indicate the prognosis of these patients [5].

Emerging evidence has demonstrated the role of radiotherapy in augmenting the efficacy of immunotherapy. However, due to the lack of laser focus, radiotherapy inevitably damages both the tumor tissue as well as the adjacent tissue, resulting in undesirable side effects. Kramer-Marek’s group from the UK presented an interesting study that incorporated targeted therapy, radiotherapy, and immunotherapy. Specifically, in this study, the authors developed a novel EGFR-specific affibody molecule, which was conjugated to the photosensitizer dye IR700. This molecule enabled the authors to target EGFR in preclinical models of glioblastoma (GBM) [6]. More intriguingly, it showed that this EGFR-targeted photoimmunotherapy exhibited a promising anti-tumor effect in GBM by turning an immune-cold microenvironment into an immune-hot microenvironment. Together, these findings indicated that combination therapy holds the promise to overcome therapy resistance.

The targeted therapies mentioned above have been approved for patients with specific genomic alterations, such as ALK amplification or ROS1 fusion. Several other therapeutic targets have also showed promising results in preclinical models or in early-phase clinical trials. Müller et al. identified MCL-1 and BCL-xL as biomarkers for survival and potential targets for the treatment of thymomas and thymic carcinomas [7]. Indeed, the development of next-generation sequencing has advanced our understanding on the molecular heterogeneity of cancers. Emerging molecular subtypes have been identified, which are critical for the implementation of precision oncology. The studies from He and Zu’s group examined the molecular profiles of regulators for epitranscriptomic and epigenomic modifications. He’s group examined the transcriptomic profiles of N6-methyladenosine (m^6^A) regulators, the most abundant RNA modification, and established a model named “m^6^A score.” Based on this model, they can stratify small-cell lung cancer (SCLC) patients into high-risk and low-risk groups. They identified the m^6^A score as an independent risk factor, which was associated with resistance to adjuvant chemotherapy and immunotherapy in SCLC [8]. This is consistent to previous studies showing that the patterns of m^6^A regulators are associated with immune evasion and prognosis in other cancer types. Zu’s group evaluated the expression landscape of regulators for 5-methylcytosine (5mC), an epigenetic modification [9]. They identified two clusters of 5mC regulators that were correlated with the classical molecular subtypes and prognosis of bladder cancer. Furthermore, they established the 5mC score, an elegant algorithm that can serve as an indicator for treatment response of immunotherapy in bladder cancer. Both studies identified potential indicators for treatment response to immunotherapy. Combination of targeted therapies and immunotherapy may synergistically improve treatment outcomes for cancers.

## Conclusion

In summary, targeted therapies have changed the cancer treatment paradigm. But many patients develop treatment resistance and ultimately succumb to tumor progression. The articles in this special collection highlighted the progress and current challenges on targeted therapies, which have achieved extraordinary success in a subset of cancers with actionable targets, such as ALK and ROS1 in NSCLC. The contributing authors are leading experts on each specific topic and should be congratulated on their outstanding discoveries, which may translate into clinical trials and benefit patients in near future. More studies are warranted to advance our understanding on the mechanisms of intrinsic and acquired resistance of these targeted therapies. Combination therapies that work on distinct mechanisms of action could be a promising strategy to minimize treatment resistance. Analysis of molecular subtypes and actionable targets, along with the identification of novel biomarkers, will be key to the development of optimal treatment strategies for patients with these lethal malignancies. Last but not the least, we would like to conclude by thanking the reviewers and the editorial staff for their tremendous effort to put together this special collection.

## Abbreviations

5mC: 5-Methylcytosine
ALK: Anaplastic lymphoma kinase
EGFR: Epidermal growth factor receptor
GBM: Glioblastoma
m6A: N6-methyladenosine
NSCLC: Non-small-cell lung cancer
SCLC: Small-cell lung cancer
TKIs: Tyrosine kinase inhibitors
